# Participatory Action Research Applied to an Ear, Nose, and Throat Specialty Service Redesign in Remote Australia: A Mixed-Methods Study of Key Stakeholder Perspectives

**DOI:** 10.3390/ijerph18010167

**Published:** 2020-12-29

**Authors:** Susan P. Jacups, Irina Kinchin, Layla Edwards

**Affiliations:** 1 School of Public Health, University of Queensland, Herston, QLD 4006, Australia; s.jacups@uq.edu.au; 2 The Cairns Institute, James Cook University, Smithfield, QLD 4879, Australia; 3 Centre for Health Economics Research and Evaluation (CHERE), University of Technology Sydney, Ultimo NSW 2007, Australia; kinchini@tcd.ie; 4 School of Medicine, Trinity College Dublin, the University of Dublin, College Green, Dublin 2, Ireland; 5 Centre for Improving Palliative, Aged and Chronic Care through Clinical Research and Translation (IMPACCT), University of Technology Sydney, Ultimo, NSW 2007, Australia

**Keywords:** participatory action research, service redesign, health promotion, ear, nose, throat, ear and hearing health, rural health services, indigenous health, telehealth, stakeholder perspectives, qualitative

## Abstract

This mixed-methods study reports on the key stakeholders’ perspectives on the ear, nose, and throat (ENT) service redesign in remote Australia, using a participatory action research (PAR) approach. A primary health care (PHC) clinician survey was conducted to assess local needs and possible educational gaps in clinical knowledge. This was followed by an internal stakeholder forum and a follow-up survey with Torres and Cape Hospital and Health Service staff to gain their perspectives on current service delivery and table ideas for a new ENT health service model. Qualitative data were analyzed inductively and grouped in emerging themes. Quantitative data were imported into tables and analyzed descriptively. PAR allowed for input from 19 PHC clinicians, 10 face-to-face stakeholders perspectives, and 18 stakeholder follow-up survey respondents. Four themes emerged: 1. Training for health workers in ENT management; 2. Improved local service access; 3. New referral pathways to improve continuity of care; and 4. Introduction of telehealth. PAR engaged key stakeholders, identifying gaps in ENT service delivery, and guided the development of the new service model. The inclusion of stakeholders throughout the service redesign process is likely to create a more sustainable model of care which already has local “buy-in”.

## 1. Introduction

### 1.1. Evidence Base

**Figure 1:**
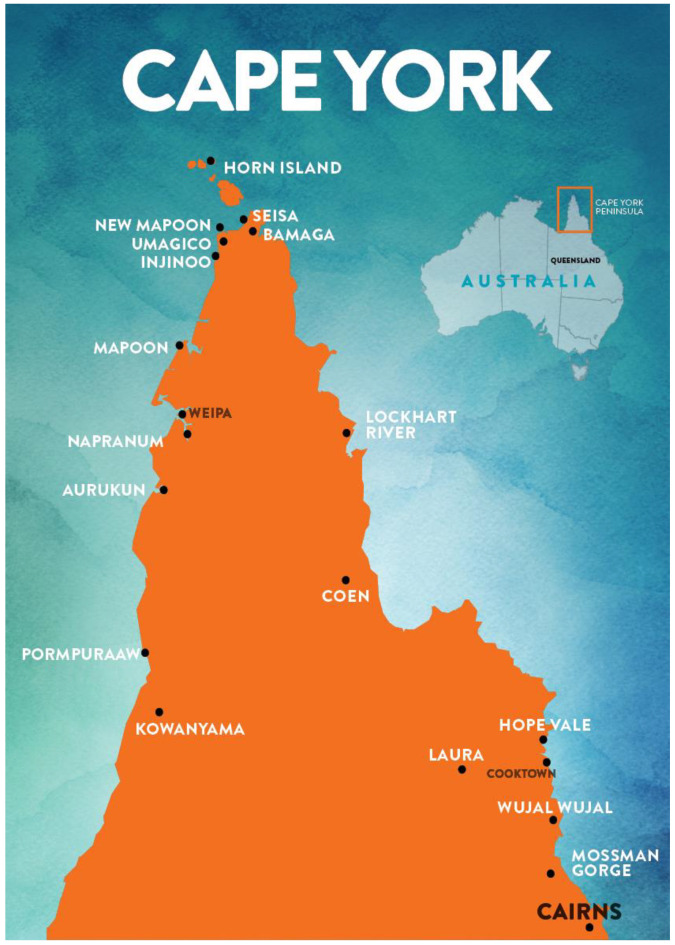
Map of Cape York Peninsula, North Queensland, Australia.

Health care delivery in rural and remote locations across Australia has established difficulties [1]. Specifically, residents of these areas experience challenges accessing health services and personal high out-of-pocket costs associated with transport to travel extensive distances [2,3]. The Cape York region in North Queensland covers an area of 113,023 square kilometers (see Figure 1). It supports a population of over 11,000 people, of whom 58% are Aboriginal or Torres Strait Islander people (hereafter, respectfully, Indigenous) [4]. Health inequalities in rural and remote settings in Australia are well researched [1,5]. The Cape York region is no exemption, with an average of 10 years lower health adjusted life expectancy than the Queensland state level [6]. In particular, the region reports high rates of otitis media (middle ear infection), often with associated hearing loss [7]. Despite this, access to ear, nose, and throat (ENT) services across the region is insufficient [7].

### 1.2. Current Service Delivery Model of Care for ENT

In 2017, specialist ENT outreach services were provided to only two (15%) Cape York communities (Wujul Wujul and Hope Vale) via the Queensland Health Deadly Ears program (Figure 1) [8,9]. People who live in the other 11 Cape York communities or townships must travel to these communities, which may include a driving distance of over 800 kilometers, to attend a face-to-face outpatient ENT specialist appointment [10]. The Deadly Ears program offers biannual ENT team visits to each community for ear and hearing screening of Indigenous children [8]. The Deadly Ears team includes specialist ENT nurses and audiologists who work alongside an ENT surgeon. In Cape York, the Deadly Ears program does not offer surgery; instead, they refer patients to the closest level 5 hospital [8]. At the time this project was undertaken, there were no outreach or telehealth services available for ENT review across Cape York, largely due to resistance within the ENT department to offer these services.

The referral hospital is challenged with long waiting times for ENT outpatient clinics and staff shortages [11]. We have previously documented the high rates of ear disease and poor hearing outcomes in this population, with ear perforations (in one or both ears) in 7% of school-aged children [7]. In addition, poor access to services has been reported, with waiting times of up to three years for elective ENT surgery [7]. Recent waiting times for children (aged 0–21) indicate breaches to categorization times [12] in over 82 percent of referrals (unpublished Queensland Health data, 2017). The Torres and Cape Hospital and Health Service employs one ENT administrator to assist and coordinate remote Cape York patient attendance at ENT outpatient appointments and surgery at the referral hospital. Both the outpatient appointments and surgery report a high fail-to-attend rate for ENT outpatient appointments, reported at 19 percent (May–July 2017, unpublished Queensland Health data).

As the current service model for ENT service delivery was insufficient to meet population needs, a service model redesign project was commissioned to create an informed new service delivery model for ENT services within Cape York. The new model aims to streamline services and build local capacity by offering local clinical ear and hearing training to improve clinician confidence when managing presenting ear and hearing conditions. This paper reports on the key stakeholders’ perspectives on the ENT service redesign, using a participatory action research (PAR) approach. Participatory action research is defined as a “collective, self-reflective inquiry that researchers and participants undertake, so they can understand and improve upon the practices in which they participate and the situations in which they find themselves. This reflective process is directly linked to action, influenced by understanding of history, culture, and local context and embedded in social relationships” [13]. Due to the ear health disparity in the area, PAR was chosen because it allows service users to provide input into defining the scope and context of the project (service redesign) and the output of the newly designed ENT service model [13,14,15]. The involvement of stakeholders in health service redesign influences the quality, dissemination, and contextualization of findings and, therefore, contributes to the sustainability of the service delivery [16]. Furthermore, PAR can improve worker satisfaction and lead to better quality outputs, which are sustained [17]. Thus, the aim of this mixed-methods study is (1) to outline primary health care clinicians’ current ENT concerns; (2) to gain ENT stakeholders’ perspectives in the current gaps and barriers to ENT services in the region; and (3) to make recommendations for a new ENT service model.

## 2. Materials and Methods

In this mixed-methods study, an initial primary health care (PHC) clinician’s survey was conducted to elicit current ENT concerns. The PHC clinician survey was followed by a stakeholder discussion forum and a follow-up stakeholder survey to thoroughly assess gaps and barriers to current ENT service delivery, and to make recommendations for a new ENT service model. The redesign project adopted a PAR framework to inform the development of a new sustainable, evidence-based model of care for ENT services across Cape York. Participation in this study was voluntary, and no additional remuneration was provided.

### 2.1. Initial PHC Staff Perspective Survey

In order to thoroughly assess local needs and possible educational gaps in clinical knowledge, a staff perspective online survey was distributed via SurveyMonkey (SVMK Inc., San Mateo, CA, USA) in January 2017. It was emailed directly to the ear or hearing PHC clinicians who provide services across Cape York from the Child and Maternal Health network group email list. At the time this survey was distributed, there were 80 members in the Child and Maternal Health network group. However, it should be noted that, due to a high turnover of staff in the region, we cannot be sure all 80 email addresses were active and received the PHC clinician survey. The survey sought information from clinicians on their level of confidence screening, diagnosing, and delivering ear and hearing treatments. The survey consisted of 15 multiple choice questions, while offering open-ended comments sections for an optional more detailed response (for full survey questions, refer to Supplementary File S1).

### 2.2. Stakeholder Open Forum and Follow-up Survey

A half-day internal stakeholder forum took place in Cairns in September 2017. Stakeholders were Torres and Cape Hospital and Health Service staff who were directly involved with clinical care or coordination (administration and data management system) of ENT services in Cape York. Potential stakeholders were recruited via email and invited to describe their views on current service delivery and identify potential solutions that could be incorporated into the development of the new ENT health service model [17]. Stakeholders were identified by the Queensland Health directory database. The forum was guided by reviewing the results from the PHC staff perspective survey, which led to stakeholders identifying gaps and barriers to current ENT service delivery. Potential solutions to identified issues were tabled, as well as alternate service delivery models. In-depth discussions led stakeholders to develop a new model which aimed to improve health and reduce health inequities for people in the Cape York region [7,18,19,20,21,22,23,24].

The forum responses formed the questions that were later offered in the follow-up stakeholder survey, which was distributed a week after the forum. The aim was to gain further input on their priorities for future ENT services. An email link to SurveyMonkey was sent to the invited forum participants, to reiterate their input and provide an opportunity to add input for those who could not attend the forum. Follow-up survey participants were further asked about the current gaps/barriers to ENT service delivery in Cape York, and what services they would like to see in an ENT outreach and surgical service within Cape York.

### 2.3. Data Collection and Analysis

The initial PHC clinician survey was analyzed prior to the forum. Quantitative data captured on the online survey were exported into, and analyzed descriptively using, Microsoft Excel. Additional comments made in the survey were extracted into a table in Microsoft Word and analyzed thematically. The forum was recorded and transcribed, verbatim, by health service administration. Handwritten notes and minutes were also taken and added to the qualitative data analysis. The follow-up survey responses were combined with the stakeholder forum data and stored in SurveyMonkey. Together, the combined qualitative data were analyzed inductively and grouped into emerging themes by two independent investigators. Any conflicting opinions among the authors were resolved through discussion.

### 2.4. Ethics

This study was reviewed by the Far North Queensland Human Research Ethics Committee and granted an exemption from full ethical review, as it meets criteria as a quality improvement activity; reference number HREC/17/QCH/3-1111 QA. Any activity where the primary purpose is to improve the quality of service delivered by an individual or organization may apply for a quality improvement exemption [25]. Therefore, participation in the survey, open forum, and follow-up survey was voluntary, and informed consent was not necessary for participation. As a result of this, no demographics of participants were collected, and qualitative data is non-identified.

## 3. Results

### 3.1. PHC Staff Perspective Survey

A total of 19 PHC clinicians responded to the initial survey. The respondents were from a range of clinical professions, including clinical nurses (42%), medical officers (16%), and indigenous health workers (11%) (see Appendix A., Table A1 for full survey responses).

#### 3.1.1. Assessing, Diagnosing, and Management of Ear Conditions

**Figure 2:**
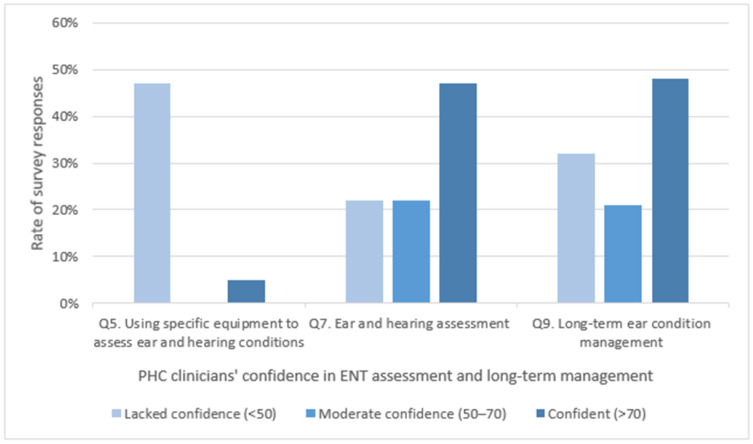
Clinician confidence at assessment, using ear-specific equipment and management of ear and hearing conditions.

Half of the respondents (44%) had limited confidence in the assessments of ear or hearing conditions (Figure 2 and Table A1). Furthermore, half of the respondents (range: 42–48%) reported they lacked confidence in using the necessary equipment to assess ear and hearing conditions. Forty-four percent (44%) of the respondents reported the highest level of uncertainty when diagnosing or assessing otitis media with effusion, and 22% of respondents felt unsure of the remaining conditions. A third (32%) of respondents also reported a lack of confidence in the long-term management of all ENT conditions. When participants were asked about the specific conditions they were not confident in managing, 25% responded otitis media with effusion, whilst the remaining conditions ranged from 13–19%.

#### 3.1.2. Telehealth

Most of the respondents (81%) reported that they considered telehealth consultations for ENT services would be well-received in the community. Additional comments for this section (see Table 1) suggested that telehealth would enable greater family involvement in health care, assisting patients in making informed decisions.

#### 3.1.3. Training and Further Education in Ear and Hearing Health

The majority (84%) of respondents said they would like to attend face-to-face ear and hearing education sessions. Furthermore, individual answers reiterated the importance of training and refresher courses to all staff, especially immediately after training to lock in newly learnt skills. The greatest level of interest (100%) was for ear suctioning and tympanometry training). Interest was also high for long-term management of otitis media conditions (80%), identification of different otitis media conditions (60%), and how and when to refer to ENT specialists (73%). Other comments made in the free text section highlighted the current issues with the available ENT services and the need to improve intra-agency communication to improve ENT services.

### 3.2. Stakeholder Forum and Follow-up Survey

Twenty key stakeholders were invited to attend the open forum, of which 10 participants (50%) attended. The follow-up survey was sent to the same 20 key stakeholders, which resulted in 18 participants (90%) completing the survey. Stakeholders included child health and public health nurses, medical officers, and system administrators. Gaps and barriers in current ENT services were identified at the forum and then presented again at the follow-up survey for agreeance (Table 2). Stakeholders identified that, due to the lack of confidence in assessing and managing ear and hearing conditions, PHC clinicians often made inappropriate referrals to specialist services (Table 2 and Table 3). As a result, these services experience long waiting times and, also, high rates of non-attendance. Another issue with referrals was that ENT specialists found that patient records would be regularly incomplete. Missing data meant specialists had to double up on services to get the essential information required for that day’s appointment, resulting in the extra time needed with patients and increasing waiting times for future patients. Telehealth is not currently offered in the area. An overburdened system and high staff turnover could be reasons for the lack of take-up of telehealth services. Potential solutions, and their feasibility were then discussed, which were used as the building blocks for the new model of care. Four themes emerged from the forum and follow-up survey: (1) training for health workers in ENT management, (2) improved local service access, (3) new referral pathways to improve continuity of care, and (4) introduction of telehealth (Table 3).

#### 3.2.1. Theme 1: Training for Health Workers in ENT Management

Stakeholders identified that the first step in improving current ENT services must include training all current and future PHC clinician staff (Table 3). Training would improve confidence in assessing and managing ear and hearing conditions, and the use of equipment needed. Additional hearing health promotion and funding is needed for this to be executed in this region.

#### 3.2.2. Theme 2: Improve Local Service Access

Limited access to ENT services is a known barrier to healthcare for the region. As a result, improving local access needs to be a priority. Stakeholders would like to see access to ENT services in all thirteen communities. Furthermore, the high turnover of staff has been identified as an issue, due to a lack of consistency. Consistency of staff has the ability to improve health outcomes for patients, as they are likely to follow healthcare directives and come back for follow-up appointments.

#### 3.2.3. Theme 3: New Referral Pathways to Improve Continuity of Care

Stakeholders identified that the recommended pathways for escalation to ENT specialist referral were inconsistent, which resulted in congested services. Patients have to travel far distances for specialist services, and, as a result, many do not show for appointments. Further, it was recognized that a lack of centralized coordination of ear health activities exacerbated the confusion around ENT care delivery, which results in frequent duplication in service. The new model of care will include a checklist for PHC clinicians, and it was recommended that regular monitoring is essential to ensure that new clinical pathways are adhered to by staff at all levels.

#### 3.2.4. Theme 4: Introduction to Telehealth

Stakeholders have identified that PHC clinicians already have the capacity to use telehealth within their practice, but uptake is poor, potentially due to a lack of resources such as time, training, and equipment. Stakeholders strongly supported the use of telehealth, as it has the ability for PHC clinicians to get second opinions or advice by sending images to specialist services or other clinicians.

## 4. Discussion

Participatory action research allowed for input from 19 PHC clinicians, 10 face-to-face stakeholders’ perspectives, and 18 stakeholder follow-up survey respondents in regard to the development of a new ENT model of care for Cape York. The aims were to outline PHC clinicians’ current ENT concerns, to gain ENT stakeholders’ perspectives in the current gaps and barriers to ENT services in the region, and to make recommendations for a new ENT service model. Findings from both surveys and the open discussion indicated stakeholders’ highest priorities were clinical education and system improvement. They outlined that the current provision of specialist ENT services upon a poorly-functioning PHC service had created dependence on the ENT specialist for routine PHC level ear and hearing concerns. Their priorities were for training and greater investment in PHC level care, with improved referral systems and greater use of telehealth for timely ear review.

The four themes that emerged from this research echoed the missing features in the current service model. These themes included the need for ongoing training, improved access to services, improved referral pathways, and greater access to telehealth, once established, that could then be applied to the revision of the service model. Previous research demonstrated that the use of telehealth has the potential to reduce waiting times by 31% in remote Alaska [7,26]. A community-based mobile telehealth screening service in remote Australia for Aboriginal and Torres Strait Islander children was found to successfully provide specialist review and treatment planning at a distance [27]. Furthermore, a Queensland telehealth scoping review identified that face-to-face consultations for ENT consultations could be reduced by 89% if telehealth were used [7,28].

The use of PAR in this study enabled service redesign that has developed a more relevant and appropriate service model than would otherwise have been developed [14,15]. As with other research in this area, PAR assists with maintaining the relationships behind the research objectives [14]. It takes the research a further step than undertaking only a literature review [15]; the practice of which has been criticized as ineffective as a sole approach to obtaining community or stakeholder involvement [15]. In combination with findings from the ENT referral review [11] and the economic costing, as previously described [29], PAR provided sufficient input for the development of a new ENT service model. It is also important to acknowledge that PAR can be selective or partial. Specifically, the use of PAR does limit the extent to which the findings from this research can be generalizable beyond this setting [30]. However, given the unique setting and population this study concerns, PAR has enabled a deeper understanding of current issues, and provided sustainable improvements for future services.

## 5. Conclusions

Participatory action research provided the avenue for stakeholder input that was sound, and their involvement in the process is more likely to create a sustainable model. Redesigned models that are tailored to local needs and to promote cross-agency collaboration are more likely to be successful and increase access to health services. This process of applying PAR methods to developing a new service delivery model may be utilized by other health services considering service redesign or revision, nationally or internationally. It is hoped that this method may act as a template for the redesign of other specialty health areas across this region.

## Figures and Tables

**Table 1:** Unprompted, additional comments made in the primary health care (PHC) clinician survey.

Survey Question	Additional Comments
Q12 Telehealth	*“Rather than travelling for a consult this prepares families to make better decision & choices for themselves about ENT services”*
Q14 and Q15 Training and Further Education	*“Need for continual training and support for staff—also new staff training”* *“Those attending [a training session] should have ear health included in their clinical practice post-training and have strategic plans for using the training”*
Free text	*“[There is a] lack of ENT service… delays in ENT services… lack of coordination of ENT services… [and] lack of evidence of ENT interventions”* *“Need to improve communication from leaders to clinicians, also, intra-agency communication between service providers regarding ENT services in the Cape. Improvements in communication will assist clinicians on the ground know what is happening and to avoid duplication of services”*

**Table 2:** Gaps and barriers to current ear and hearing service delivery identified by forum stakeholders, and the rate of agreeance by follow-up survey respondents (*n* = 18).

Identified Gaps/Barriers in Ear and Hearing Service Delivery	Rate of Agreeance by Follow-up Survey Respondents (%)
Administration systems poorly functioning, i.e., referrals, appointment processes	88%
Ear pathology difficult in PHC setting, making diagnosis by PHC clinicians poor	71%
Ear condition management not always adhered to by PHC clinicians	71%
Telehealth not well supported by PHC clinicians	53%
Specialist recommendations not adhered to by PHC clinicians	41%
Other	41%

**Table 3:** Themes, barriers, and solutions identified at stakeholder forum and follow-up survey.

Identified Gaps/Barriers in Ear and Hearing Service Delivery	Potential Solutions
Theme 1: Training for health workers in ENT management	
*“* *Poor diagnostics skills in community for ear conditions- therefore inappropriate management”*	*“More health workers and nurses properly trained at ground level”* *“Appropriate management and hearing health promotion funded more extensively and given more recognition of need”*
Theme 2: Improve local service access	
*“As a PHC GP I found that the service provision has been so infrequent that we basically have no ENT service in Cape York. Therefore all my clients are referred to Cairns which is sometimes very difficult for my patients to attend and they miss appointments”*	*“Regular visits by outreach specialists with the possibility to discuss in person regular review or follow up of the patients seen clear advice how to continue management or treatment”* *“More regular visits to build rapport with communities so that recs are more likely to be followed”*
Theme 3: New referral pathways to improve continuity of care	
*“Present referrals, appointments, telehealth, and clinical data storage, all fallible”* *“PHC care plans not being followed—Staff not recording information, such as commencement of procedures (dry mopping)… Filing of documentation, evidence of referrals, care paths, consultations etc. in patient’s medical notes”* *“Challenging getting feedback when multiple services work in one community including some duplications in service. Only one audiologist employed by Apunipima means she’s extremely stretched and there’s a delay getting results of screening tests. Difficult to get feedback on clients seen by Australian hearing re: plan for follow up etc.”*	*“A regular service that is well coordinated with systems aligning with current processes, communication of case management needs to be completed from referral, review, management, follow-up and discharge.”* *“Soon to roll out checklist which is aimed at addressing [lack of patient information]”* *“Local review & theatre—increases compliance of patients attending appointments/operations. Maintains skills of local clinicians”*
Theme 4: Introduction to telehealth	
*“[ENT] services should be supported via telehealth but with lack of appropriate clinical support, turnover of staff, need for specialist equipment etc. implementation is proving difficult at this time.”*	*“More telehealth with images sent from appropriate machines giving good images. Possibly an email link to consultants for guidance regarding management and a regular outreach audiometry service”* *“Telehealth training: take otoscopic images and send them for review…ENT/specialist who reviews them can be anywhere… Train staffed to use digital otoscope and take pictures—less reliance on diagnostic skills”*

## Data Availability

All available data is provided here, no further data is available.

## Supplementary Materials

The following are available online at https://www.mdpi.com/1660-4601/18/1/167/s1, File S1: PHC clinician survey.

## Appendix A

**Table A1:** Primary health care (PHC) clinician multiple choice survey responses.

Survey Multiple Choice Options (Number of Responses)	N (%)
Demographics of Respondents	
*Q1 Current working role in Cape York (n = 19)*	
Clinical Nurse	8 (42%)
Medical Officer	3 (16%)
Indigenous Health Worker	2 (11%)
Nursing Manager	2 (11%)
Audiologist	1 (5%)
Visiting Specialist	1 (5%)
Other (please specify)	2 (11%)
*Q2 Length in current role (n = 19)*	
≤3 months	1 (5%)
3–6 months	0 (0%)
7–12 months	1 (5%)
13 months–5 years	13 (68%)
≥6 years	4 (21%)
Community Ear and Hearing Health Process Review	
*Q3 Work for any of the following primary health care providers? (n = 17)*	
Torres and Cape Hospital Health Service	9 (53%)
Apunipima Cape York Health Council	7 (41%)
Royal Flying Doctor Service	3 (18%)
Deadly Ears Program	1 (6%)
*Q4 Equipment used to asses ear and hearing health (n = 18)*	
Otoscope	18 (100%)
Tympanometer	13 (72%)
Audiometer	6 (33%)
Video otoscope	3 (17%)
Flexicam otoscope	0 (0%)
*Q5. Lack of confidence using specific equipment (n = 19)*	
Audiometer	9 (47%)
Tympanometer	9 (47%)
Otoscope	8 (42%)
Video otoscope	8 (42%)
Flexicam otoscope	8 (42%)
None—I am confident using all equipment	1 (5%)
*Q6 Clinical care guidelines used when reviewing ear or hearing presentations (n = 19)*	
Primary clinical care manual	17 (90%)
Clinical care guidelines for treatment of otitis media in Aboriginal & Torres Strait Islander populations	8 (42%)
Clinical judgement	7 (37%)
Therapeutic guidelines	4 (21%)
Deadly Ears guidelines and protocols	2 (11%)
Central Australian Rural Practitioners Association	1 (5%)
Clinical Prioritisation Criteria	0 (0%)
*Q7 Confidence in making ear/hearing assessments (n = 18)*	
Not confident (<20)	2 (11%)
Not very confident (20–50)	2 (11%)
A little confident (51–70)	4 (22%)
Quite confident (71–90)	7 (40%)
Very confident (>90)	3 (7%)
*Q8 Conditions you’re unsure of in your ear assessments (n = 18)*	
Otitis media effusion	8 (44%)
Acute otitis media	4 (22%)
Acute otitis media without perforation	4 (22%)
Acute otitis media with perforation	4 (22%)
Chronic suppurative otitis media	4 (22%)
Dry perforation	4 (22%)
None—confident in all assessments	6 (33%)
Other (exostosis/osteoma *n* = 1; early cholesteatoma *n* = 1)	2 (11%)
*Q9 Confidence in long-term ear condition management (n = 19)*	
Not confident (<20)	3 (16%)
Not very confident (20–50)	3 (16%)
A little confident (51–70)	4 (21%)
Quite confident (71–90)	6 (32%)
Very confident (>90)	3 (16%)
*Q10 Lack of confidence in managing conditions (n = 16)*	
Otitis media with effusion	4 (25%)
Acute otitis media with perforation	3 (19%)
Chronic suppurative otitis media	3 (19%)
Dry perforation	3 (19%)
Acute otitis media	2 (13%)
Acute otitis media without perforation	2 (13%)
None—I’m confident in all of them	8 (50%)
*Q11 Reasons for referral to medical or ENT specialist for advice (n = 19)*	
To refer for surgery	15 (79%)
For a second opinion on a clinical decision	14 (74%)
To take over patient ear condition management	12 (63%)
For ear condition management advice	12 (63%)
Other (if not seen in timely manner)	1 (5%)
*Q12 Telehealth for ENT consults (n = 16)*	
I consider they would be well received	13 (81%)
No—I don’t think they would be well received	3 (19%)
*Q13 Would community members (patient/carers) welcome Ear Health information to take home? (n = 17)*	
I consider this would be well received	10 (83%)
No—I don’t consider they would be well received	2 (17%)
*Q14 Face-to-face ear and hearing training for PHC clinicians (n = 19)*	
Yes	16 (84%)
No	0 (0%)
Other	3 (16%)
*Q15 Specifically, what training interests you (n = 15)*	
Ear suctioning and tympanometry	15 (100%)
Long-term community management of otitis media	12 (80%)
How and when to seek specialist ENT input (referral)	11 (73%)
Use of current guidelines for treatment and management of ear disease	11 (73%)
Identification of different otitis media conditions	9 (60%)
All of the above	2 (13%)
