# Supplementary material for: Participatory Action Research Applied to an Ear, Nose, and Throat Specialty Service Redesign in Remote Australia: A Mixed-Methods Study of Key Stakeholder Perspectives
Source: Int J Environ Res Public Health. 2020 Dec 29;18(1):167. doi: 10.3390/ijerph18010167 (PMC7796165; doi:10.3390/ijerph18010167)
Supplement: Supplementary file 1 [file ijerph-18-00167-s001.pdf]

Community Ear/hearing health process review

Ear health clinical management

1. What best describes your working role in Cape York Primary Health Centres?

- Indigenous Health Worker
- Clinical Nurse
- Nursing Manager
- Audiologist
- Medical Officer
- Visiting Specialist
- Other (please specify)

2. How long have you worked in this role (or similar role at another location)?

- < 3months
- 3-6 months
- 6-12 months
- > 12 months
- Other (please specify)

3. Do you work for any of the following Primary Health Care providers?

- [ ] Apunipima Cape York Health Council
- [ ] Royal Flying Doctor Service (RFDS)
- [ ] Torres and Cape Hospital Health Service (TCHHS)
- [ ] Deadly Ears Program - Queensland Health
- [ ] Other (please specify)

4. When you assess ears or hearing health, what equipment do you use?

- [ ] Otoscope
- [ ] Video otoscope
- [ ] Flexicam Otoscope
- [ ] Tympanometer
- [ ] Audiometer
- [ ] Australian Hearing App- Hearing scouts
- [ ] Other (please specify)

5. Which of these pieces of equipment, if any, you do not feel confident using?

- [ ] Otoscope
- [ ] Video otoscope
- [ ] Flexicam Otoscope
- [ ] Tympanometer
- [ ] Audiometer
- [ ] Australian Hearing App- Hearing scouts
- [ ] All of the above
- [ ] None- I am confident using all equipment
- [ ] Other (please specify)

6. Which clinical care guidelines do you mostly use when reviewing Ear/ hearing presentations?

- [ ] Primary Clinical Care Manual (PCCM-QH)
- [ ] Clinical Care guidelines for treatment of Otitis Media in Aboriginal & Torres Strait Islander populations (NHMRC)
- [ ] Therapeutic Guidelines
- [ ] Clinical Prioritisation Criteria (QHEPS-QH)
- [ ] Deadly Ears Guidelines and Protocols- QH
- [ ] Clinical judgement
- [ ] CARPA
- [ ] Other (please specify)

7. How confident do you feel about making Ear/hearing assessments?

Not confident **Confident**

8. Which of the following conditions, if any, are you unsure of in your ear assessments?

- [ ] Acute Otitis Media (AOM)
- [ ] Acute Otitis Media without Perf (AOMwoP)
- [ ] Acute Otitis Media with Perf (AOMwiP)
- [ ] Chronic suppurative Otitis Media (CSOM)
- [ ] Dry Perforation
- [ ] Otitis Media Effusion (OME)
- [ ] none - I am confident in all of them
- [ ] Other (please specify)

9. How confident do you feel about long-term Ear condition management?

not very confident **confident**

10. Which conditions, if any, do you not feel confident managing?

- [ ] Acute Otitis Media (AOM)
- [ ] Acute Otitis Media without Perf (AOMwoP)
- [ ] Acute Otitis Media with Perf (AOMwiP)
- [ ] Chronic suppurative Otitis Media (CSOM)
- [ ] Dry Perforation
- [ ] Otitis Media Effusion (OME)
- [ ] None- I'm confident in all of them
- [ ] Other (please specify)

11. Which of these reasons would you refer for medical advice or ENT specialist advice?

- [ ] For a second opinion on a clinical decision
- [ ] To take over patient Ear condition management
- [ ] For Ear condition management advice
- [ ] To refer for surgery
- [ ] Other (please specify)

12. How do you think using TeleHealth for ENT consults would be received in your community?

- ( ) I consider they would be well received
- ( ) No- I don't think they would be well received

Reason for this response

13. Would community members (patient carers) welcome Ear Health information to take home?

- ( ) I consider this would be well received
- ( ) No- I don't consider they would be well received

Reason for your response

14. Would you be happy to attend face-to-face Ear and hearing training?

- ◯ Yes
- ◯ No, thanks
- ◯ Other (please specify)

[ ]

15. Which of the following, if any, would you be happy to attend?

- ☐ Identification of different Otitis Media conditions
- ☐ Use of current guidelines for treatment and management of ear disease
- ☐ Ear suctioning and tympanometry
- ☐ Long-term community management of Otitis Media conditions
- ☐ How and when to seek specialist ENT input (referral)

Other (please specify)

[ ]

16. Please let us know of other ENT issues you are concerned about

[ ]

17. If you would like further information on training or to be involved in discussions on ENT model of care development, please provide your contact details below:

Name [ ]

Email Address [ ]

Phone Number [ ]
